# Synthesis of [^11^C]carbonyl-labeled cyclohexyl (5-(2-acetamidobenzo[*d*]thiazol-6-yl)-2-methylpyridin-3-yl)carbamate ([^11^C-carbonyl]PK68) as a potential PET tracer for receptor-interacting protein 1 kinase

**DOI:** 10.1186/s41181-022-00156-1

**Published:** 2022-03-15

**Authors:** Tomoteru Yamasaki, Katsushi Kumata, Atsuto Hiraishi, Yiding Zhang, Hidekatsu Wakizaka, Yusuke Kurihara, Nobuki Nengaki, Ming-Rong Zhang

**Affiliations:** 1 Department of Advanced Nuclear Medicine Sciences, Institute for Quantum Medical Science, Quantum Life and Medical Science Directorate, National Institutes for Quantum Science and Technology, 4-9-1 Anagawa, Inage-ku, Chiba, 263-8555 Japan; 2 SHI Accelerator Service Co. Ltd., 1-17-6 Osaki, Shinagawa-ku, Tokyo, 141-0032 Japan

**Keywords:** PK68, PET, RIPK1, [^11^C]acetyl chloride

## Abstract

**Background:**

Receptor-interacting protein 1 kinase (RIPK1) is a key enzyme in the regulation of cellular necroptosis. Recently, cyclohexyl (5-(2-acetamidobenzo[*d*]thiazol-6-yl)-2-methylpyridin-3-yl)carbamate (PK68, **5**) has been developed as a potent inhibitor of RIPK1. Herein, we synthesized [^11^C]carbonyl-labeled PK68 ([^11^C-carbonyl]PK68, [^11^C]PK68) as a potential PET tracer for imaging RIPK1 and evaluated its brain uptake in vivo*.*

**Results:**

We synthesized [^11^C]PK68 by reacting amine precursor **14** with [^11^C]acetyl chloride. At the end of synthesis, we obtained [^11^C]PK68 of 1200–1790 MBq with a radiochemical yield of 9.1 ± 5.9% (n = 10, decay-corrected to the end of irradiation) and radiochemical purity of > 99%, and a molar activity of 37–99 GBq/μmol starting from 18–33 GBq of [^11^C]CO_2_. The fully automated synthesis took 30 min from the end of irradiation. In a small-animal PET study, [^11^C]PK68 was rapidly distributed in the liver and kidneys of healthy mice after injection, and subsequently cleared from their bodies via hepatobiliary excretion and the intestinal reuptake pathway. Although there was no obvious specific binding of RIPK1 in the PET study, [^11^C]PK68 demonstrated relatively high stability in vivo and provided useful structural information further candidate development.

**Conclusions:**

In the present study, we successfully radiosynthesized [^11^C]PK68 as a potential PET tracer and evaluated its brain uptake. We are planning to optimize the chemical structure of [^11^C]PK68 and conduct further PET studies on it using pathological models.

## Background

Receptor-interacting protein 1 kinase (RIPK1) is a key regulator of neuronal death and is involved in apoptosis and necroptosis; it is related to several disorders including neuroinflammation, neurodegeneration, carcinogenesis, and liver inflammation (Chan et al. 2019; Brenner et al. 2013; DeRoo et al. 2020; Yu et al. 2021; Yuan et al. 2019). RIPK1 is a member of the RIP kinase family and contains a Ser/Thr kinase domain N-terminal, an intermediate domain, and a death domain C-terminal (Zhang et al. 2018a). The death domain of RIPK1 binds to tumor-necrosis factor-associated proteins related to cellular death. In addition, the intermediate and death domains of RIPK1 enable it to form with a variety of other kinases, such as RIPK3, focal adhesion kinase, and mitogen-activated protein/extracellular signal-regulated kinases (Zhang et al. 2010). Therefore, it is supposed that RIPK1 plays a crucial role in regulating cell death and controlling the homeostasis of tissues and organs and abundantly exists in tissues with intense cellular metabolism, such as intestine and skin (Kaczmarek et al. 2013). Although expression level of RIPK1 in the brain has never been reported until now, it was reported that mRNA level of RIPK1 in healthy control was expressed with same degree as that of glyceraldehyde-3-phosphate dehydrogenase (GAPDH) (Jayaraman et al. 2021). Additionally, in that report, mRNA level of RIPK1 increased about two-folds in the Alzheimer’s disease patients compared with healthy control. For cyclooxygenase-2 (COX-2), a biomarker for neuroinflammation in positron emission tomography (PET) imaging, the mRNA of COX-2 in activated microglial cells induced by administration with lipopolysaccharide was expressed with similar degree to GAPDH (Nam et al 2018), it was therefore assumed that expression level of RIPK1 would be sufficient in brain tissue under the normal or pathologic conditions, making RIPK1 visible by PET.

**Fig. 1:**
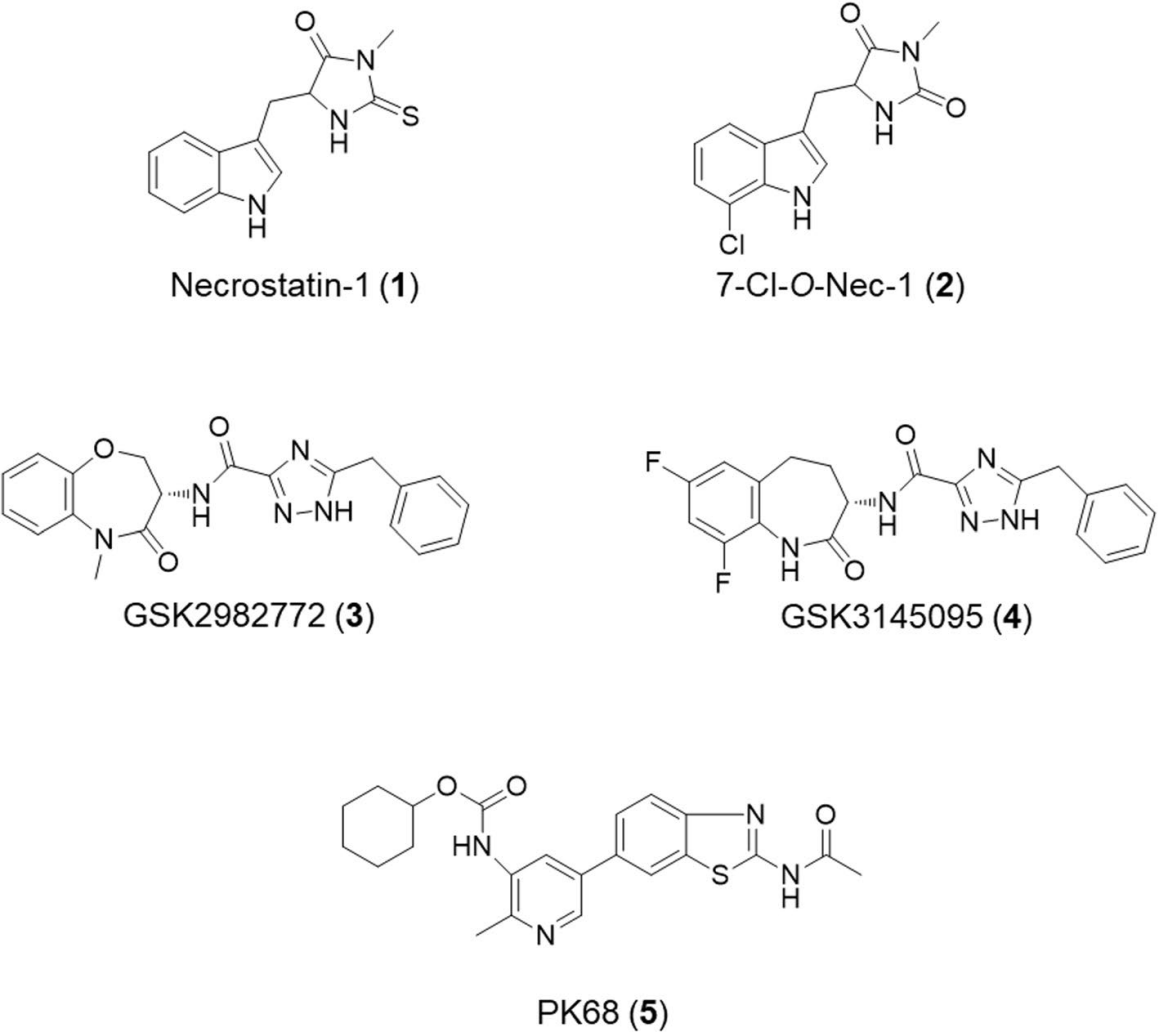
Chemical structures of receptor-interacting protein 1 kinase (RIPK1) inhibitors

Several RIPK1 inhibitors have been developed, as shown in Fig. 1. Necrostatin-1 (**1**) and its derivative (**2**), which is also called 7-Cl-*O*-Nec-1, were initially identified as allosteric RIPK1 inhibitors (Degterev et al. 2005). However, the half maximal effective concentrations (EC_50_ values) of these compounds were 490 nM for **1** and 210 nM for **2**, which would be insufficient for PET imaging of RIPK1. Recently, two different types of potent RIPK1 inhibitors have been identified (Fig. 1) (Harris et al. 2017; Hou et al. 2019). GlaxoSmithKline (GSK) inhibitors are extremely potent in human cells (IC_50_ = 6.3 nM for **3** and IC_50_ = 10 nM for **4**), and have been used in clinical trials of treatments for inflammatory diseases and central nervous system disorders (Weisel et al. 2017; Yuan et al. 2019). However, the efficacy of these compounds is highly reduced in rodents (IC_50_ >1 μM for **3** and IC_50_ >3 μM for **4**) (Harris et al. 2017, 2019). Although cyclohexyl (5-(2-acetamidobenzo[*d*]thiazol-6-yl)-2-methylpyridin-3-yl)carbamate (PK68, **5**) has lower efficacy than GSK compounds with regard to RIPK1 in human cell lines, it does not exhibit species selectivity (IC_50_ = 23 nM for a human cell line and IC_50_ = 13 nM for a mouse cell line) (Hou et al. 2019).

**Fig. 2:**
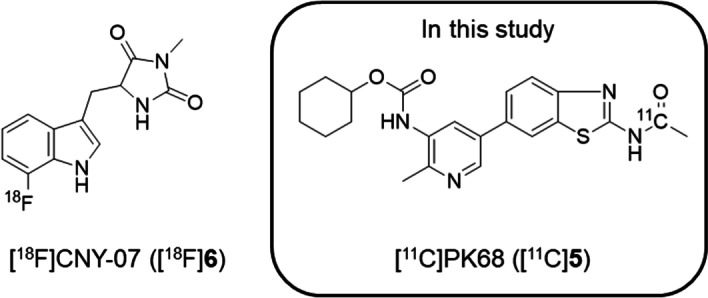
Chemical structures of current radiotracers for imaging receptor-interacting protein 1 kinase (RIPK1)

PET is an advanced molecular imaging modality that is widely used to evaluate distribution in vivo and to measure the target occupancy of pharmaceuticals using small animals (Saijo et al. 2009; Yamasaki et al. 2020). Most recently, [^18^F]CNY-07 ([^18^F]**6**), an analog of 7-Cl-*O*-Nec-1, has been developed as an initial PET tracer for imaging RIPK1 (Lan et al. 2021) (Fig. 2). Although [^18^F]CNY-07 exhibited relatively high affinity (K_D_ = 68 nM for RIPK1), high nonspecific binding and slow clearance from brain may hamper in vivo visualization of RIPK1 in brain using PET with [^18^F]CNY-07. Therefore, to the best of our knowledge no clinically useful PET tracers that can be used to visualize RIPK1 in vivo have been developed until now. Herein, we radiolabeled PK68, a potent RIPK1 inhibitor without species selectivity, with ^11^C via [^11^C]acetyl chloride ([^11^C]AcCl), and preliminary evaluated the brain uptake of [^11^C-carbony]PK68 ([^11^C]PK68, [^11^C]**5**) as a potential PET tracer.

## Methods

### General

All chemical reagents and organic solvents were purchased from FUJIFILM Wako Pure Chemicals (Osaka, Japan), Tokyo Chemical Industries (Tokyo, Japan), Nacalai Tesque (Kyoto, Japan), and BLD Pharmatech (Shanghai, China), and were used without further purification. Proton nuclear magnetic resonance (^1^H-NMR) and carbon 13 nuclear magnetic resonance (^13^C-NMR) spectra were recorded on ECS-400 (JEOL, Tokyo, Japan), ECA-500 (JEOL), and ECZ-600R (JEOL) spectrometers. The chemical shifts of the ^1^H-NMR and ^13^C-NMR spectra were reported as δ values (ppm) relative to tetramethyl silane (0 ppm) and relative to CDCl_3_ (77.0 ppm) or dimethyl sulfoxide (DMSO)-*d*_6_ (39.6 ppm). The splitting patterns were reported as s (singlet); d (doublet); t (triplet); q (quartet); m (multiplet); and br (broad). The coupling constants (J values) are given in hertz (Hz). The electrospray ionization–mass spectrometry (ESI–MS) spectra were recorded on a Q-Exactive Plus spectrometer (Thermo Scientific, Waltham, MA, USA). High-resolution fast atom bombardment mass spectra (HRMS) were acquired using an NMS-SX102 102A spectrometer (JEOL). Column chromatography was performed using Wako-Gel C-200 (100–200 mesh). The purities of the synthesized compounds for biological testing were > 98% as determined by analytical high-performance liquid chromatography (HPLC). Unless otherwise stated, radioactivity was measured using an IGC-3R Curiemeter (Hitachi Aloka Medical, Tokyo, Japan). HPLC was performed using a JASCO HPLC system (JASCO, Tokyo, Japan): effluent radioactivity was monitored using a NaI (Tl) scintillation detector system.

### Chemical synthesis

#### 6-(4,4,5,5-Tetramethyl-1,3,2-dioxaborolan-2-yl)-2-amino benzo[d]thiazole (8)

A mixture comprising 2-amino-6-bromobenzothiazole (**7**; 2.29 g, 10.0 mmol), bis(pinacolato)diboron (2.79 g, 11.0 mmol), K_2_CO_3_ (2.94 g, 30 mmol), and Pd(dppf)_2_ dichloromethane adduct (0.8 g, 1.0 mmol) in 1,4-dioxane (40 mL) was refluxed under argon for 10 h. The reaction mixture was filtered through a celite bed and washed with AcOEt. After solvent removal, the residue was purified by column chromatography (silica gel, CH_2_Cl_2_/AcOEt = 100/0 to 80/20) to give **8** as a white powder (2.06 g) with a melting point (mpt) of 218–219 °C (recrystallized form *n*-hexane). ^1^H-NMR (400 MHz, CDCl_3_) δ 8.06 (1H, s), 7.75 (1H, dd, *J* = 0.9, 9.0 Hz), 7.52 (1H, d, *J* = 8.1 Hz), 5.59 (2H, br), 1.35 (12H, s). ^13^C-NMR (151 MHz, CDCl_3_) δ 167.31, 154.47, 132.43, 131.24, 127.70, 118.51, 83.78, 31.57, 24.86. MS (ESI) [M + H]^+^ calculated for (C_19_H_17_BN_2_O_2_S) requires *m/z*, 277.1177; found *m/z*, 277.1170.

#### 2-Acetylamino 6-(4,4,5,5-tetramethyl-1,3,2-dioxaborolan)benzo[d]thiazole (9a)

Acetylchloride (0.4 mL, 6.0 mml) in CH_2_Cl_2_ (2.0 mL) was added to a mixture comprising **8** (1.10 g, 4.0 mmol), Et_3_N (1.39 mL, 10.0 mmol), and *N*,*N*-dimethylaminopyridine (DMAP) (15 mg, 0.12 mmol) in CH_2_Cl_2_ (8 mL), which was stirred at room temperature overnight. The reaction was quenched by brine and extracted with CH_2_Cl_2_. The organic layer was washed with brine, dried over sodium sulfate, and evaporated. The residue was purified by column chromatography (silica gel, *n*-hexane/AcOEt = 90/10 to 80/20) to give **9a** as a white powder (0.96 g) with a mpt of 235–236 °C (decomp). ^1^H-NMR (400 MHz, DMSO-*d*_6_) δ: 12.45 (1H, brs), 8.27 (1H, s), 7.72 (2H, s), 2.21 (3H, s), 1.31 (12H, s). ^13^C-NMR (125.7 MHz, CDCl_3_): δ 168.8, 150.0, 149.9, 132.5, 131.4, 128.6, 125.8, 119.6, 83.0, 24.9, 23.5. MS (FAB) [M + H]^+^ *m/z*, 319.

#### *tert*-Butyl 6-(4,4,5,5-tetramethyl-1,3,2-dioxaborolan-2-yl)benzo[d]thiazol-2-yl-carbamate (9b)

Di-*tert*-butyl dicarbonate (1.64 g, 7.5 mmol) was added to a mixture comprising **7** (2.0 g. 7.24 mmol), Et_3_N (1.39 mL, 10.0 mmol), and DMAP (30 mg, 0.25 mmol) in CH_2_Cl_2_ (25 mL), which was stirred at room temperature overnight. The reaction mixture was quenched with brine and extracted with CH_2_Cl_2_. The organic layer was washed with brine, dried over sodium sulfate, and evaporated. The residue was purified by column chromatography (silica gel, *n*-hexane/AcOEt = 90/10 to 80/20) to give **9b** as a white powder (2.17 g) with a mpt > 350 °C (decomp.) ^1^H-NMR (400 MHz, CDCl_3_): δ 11.77 (1H, br), 8.26 (1H, s), 7.91 (1H, d, *J* = 8.1 Hz), 7.82 (1H, dd, *J* = 0.9, 8.1 Hz), 1.60 (9H, s), 1.38 (12H, s). ^13^C-NMR (151 MHz, CDCl_3_): δ 163.02, 152.84, 150.97, 131.86, 131.08, 128.00, 120.08, 83.94, 83.39, 28.39, 24.86. HRMS (ESI) [M + H]^+^ calculated for (C_18_H_26_BN_2_O_4_S) requires *m/z*, 377.1701; found *m/z*, 377.1695.

### Cyclohexyl 4-nitrophenyl carbonate (11)

A mixture comprising 4-nitrophenyl chloroformate (**10**; 6.05 g, 30.0 mmol), cyclohexanol (3.30 g, 33.0 mmol), and DMAP (0.36 g, 3.0 mmol) in tetrahydrofuran (THF) (15 mL) was stirred at 0 °C for 3 h and then at room temperature for 3 h. The reaction mixture was diluted with *n*-hexane and purified by column chromatography (silica gel, *n*-hexane/AcOEt = 95/5) to give **11** as a white powder (3.83 g) with a mpt of 63–64 °C. ^1^H-NMR (400 MHz, CDCl_3_): δ 8.28 (2H, d, *J* = 9.2 Hz), 7.39 (2H, d, *J* = 9.2 Hz), 4.73–4.80 (1H, m), 1.99–2.05 (2H, m), 1.79–1.84 (2H, m), 1.55–1.64 (4H, m), 1.26–1.47 (2H, m). ^13^C-NMR (151 MHz, CDCl_3_): δ 155.66, 151.83, 145.20, 125.23, 121.78, 78.71, 31.33, 25.06, 23.50. MS (FAB) [M + H]^+^ *m/z*, 266.

### Cyclohexyl (5-bromo-2-methylpyridine-3-yl) carbamate (12)

A solution of potassium bis(trimethylsilyl)amide (5.6 mmol) in toluene (0.5 M, 11.2 mL) was added slowly to the mixture of 2-methyl-3-amino-5-bromopyridine (0.5 g, 2.67 mmol) in dry THF (5 mL) at 0 °C. After stirring for 15 min at 0 °C, a solution of **11** (0.85 g, 3.2 mmol) in THF (5 mL) was added slowly to the mixture described above. The reaction mixture was stirred for 30 min at 0 °C. The mixture was quenched with brine and extracted with AcOEt. The organic layer was washed with brine, dried over MgSO_4_, and then evaporated. The residue was purified by column chromatography on silica gel (*n*-hexane/AcOEt = 90/10 to 80/20) to give **12** as a pale-yellow powder (0.63 g) with a mpt of 95–96 °C. ^1^H-NMR (400 MHz, CDCl_3_): δ 8.48 (1H, br), 8.26 (1H, d, *J* = 2.0 Hz), 6.43 (1H, s), 4.76 (1H, m), 2.46 (3H, s), 1.74–1.97 (4H, m), 1.22–1.60 (6H, m). ^13^C-NMR (151 MHz, CDCl_3_): δ 152.82, 144.25, 133.67, 129.02, 118.30, 115.63, 74.74, 31.82, 25.24, 23.78, 20.26. MS (FAB) [M + H]^+^ *m/z*, 313.

### Cyclohexyl 5-(2-acetylamino-benzo[d]thiazol-6-yl)-2-methylpyridin-3-ylcarbamate (PK68, 5)

A mixture comprising **12** (478 mg, 1.5 mmol), **9a** (376 mg, 1.0 mmol), K_2_CO_3_ (520 mg, 3.75 mmol), and Pd(Ph_3_P)_4_ (173 mg, 0.15 mmol) in 1,4-dioxane/H_2_O (15 mL/3 mL) was refluxed under argon for 12 h. The reaction mixture was filtered through a celite bed and washed with AcOEt. The crude product was extracted with AcOEt, and the organic layer was washed with brine and dried over MgSO_4_, then evaporated. The residue was purified by column chromatography on silica gel (CH_2_Cl_2_/CH_3_OH = 100/0 to 90/10) to give PK68 (**5**) as a pale-yellow powder (440 mg) with a mpt >350 °C (decomp). ^1^H-NMR (400 MHz, DMSO-*d*_6_): δ 9.14 (1H, s), 8.59 (1H, d, *J* = 2.0 Hz), 8.30 (1H, d, *J* = 1.6 Hz), 8.10 (1H, d, *J* = 1.8 Hz), 7.81 (1H, d, *J* = 8.5 Hz), 7.71 (1H, dd, *J* = 1.9, 8.4 Hz), 4.62–4.68 (1H, m), 3.45 (1H, br), 2.46 (3H, s), 2.21 (3H, s), 1.91–1.94 (2H, m), 1.71–1.74 (2H, m), 1.21–1.52 (6H, m). ^13^C-NMR (125.7 MHz, DMSO-*d*_6_): δ 169.5, 158.6, 154.0, 150.3, 148.4, 142.6, 133.3, 132.9, 132.6, 132.2, 129.0, 120.9, 119.7, 72.9, 31.6, 24.9, 23.5, 22.8, 20.7. HRMS (FAB) [M + H]^+^ calculated for (C_22_H_25_N_4_O_3_S) requires *m/z*, 425.1647; found *m/z*, 425.1652.

### Cyclohexyl 5-(2-tert-butoxycarbonyl-aminobenzo[d]thiazol-6-yl)-2-methylpyridin-3-ylcarbamate (13)

A mixture comprising **12** (313 mg, 1.0 mmol), **9b** (376 mg, 1.0 mmol), K_2_CO_3_ (210 mg, 1.5 mmol), and Pd(Ph_3_P)_4_ (116 mg, 0.1 mmol) in 1,4-dioxane/H_2_O (10 mL/2 mL) was refluxed under argon for 12 h. The reaction mixture was filtered through a celite bed and washed with AcOEt. The crude product was extracted with AcOEt and the organic layer was washed with brine, dried over MgSO_4_, and then evaporated. The residue was purified by column chromatography on silica gel (CH_2_Cl_2_/CH_3_OH = 100/0 to 90/10) to give **13** as a pale-yellow powder (206 mg) with a mpt >350 °C (decomp). ^1^H-NMR (400 MHz, DMSO-*d*_6_): δ 11.87 (1H, br), 9.14 (1H, s), 8.59 (1H, d, *J* = 2.0 Hz), 8.28 (1H, d, *J* = 1.8 Hz), 8.10 (1H, s), 7.76 (1H, d, *J* = 8.3 Hz), 7.69 (1H, d, *J* = 10.1 Hz), 4.62–4.68 (1H, m), 2.46 (3H, s), 1.91–1.99 (2H, m), 1.72–1.74 (2H, m), 1.53 (9H, s), 1.29–1.45 (6H, m). ^13^C-NMR (151 MHz, DMSO-*d*_6_) δ: 160.2, 153.6, 150.2, 149.2, 142.6, 133.3, 132.9, 132.6, 131.8, 131.5, 128.8, 124.8, 120.6, 119.5, 81.8, 72.6, 31.6, 27.9, 24.9, 23.4, 20.7. HRMS (ESI) [M + H]^+^ calculated for (C_25_H_31_N_4_O_4_S) requires *m/z*, 483.2066; found *m/z*, 483.2055.

### Cyclohexyl-5-(2-aminobenzo[*d*]thiazol-6-yl)-2-methylpyridin-3-ylcarbamate (14)

A mixture comprising **13** (100 mg, 0.21 mmol in CH_3_OH (10 mL) and 2 mol/L HCl (5 mL) was stirred at 60 °C overnight. Et_3_N (1.5 mL) was added to the reaction mixture and evaporated. The crude product was washed with water and purified by column chromatography (silica gel CH_2_Cl_2_/CH_3_OH = 100/0 to 97/3) to give **14** as a white powder (44.3 mg) with a mpt >350 °C (decomp). ^1^H-NMR (400 MHz, DMSO-*d*_*6*_): δ 9.10 (1H, s), 8.53 (1H, d, *J* = 1.8 Hz), 8.02 (2H, d, *J* = 11.9 Hz), 7.61 (2H, br), 7.51 (1H, dd, *J* = 1.5, 8.2 Hz), 7.41 (1H, d, *J* = 8.3 Hz), 4.61–4.67 (1H, m), 2.44 (3H, s), 1.90–1.93 (2H, m), 1.72–1.77 (2H, m), 1.16–1.48 (6H, m). ^13^C-NMR (151 MHz, DMSO-*d*_*6*_): δ 167.14, 153.93, 152.78, 149.68, 142.26, 133.51, 132.77, 132.09, 129.46, 124.16, 118.92, 118.01, 72.78, 40.03, 24.88, 23.42, 20.64. HRMS (ESI) [M + H]^+^ calculated for C_20_H_22_N_4_O_2_S) requires *m/z*, 383.1542; found *m/z*, 383.1531.

## Radiochemistry

### Cyclohexyl (5-(2-[^11^C-carbonyl]acetamidobenzo[***d***]thiazol-6-yl)-2-methylpyridin-3-yl)carbamate ([^11^C]PK68)

^11^C was produced by the ^14^N (p, α)^11^C nuclear reaction using a CYPRIS HM-18 cyclotron (Sumitomo Heavy Industry, Tokyo, Japan). An automated multi-purpose synthesizer developed in-house (Fukumura et al. 2007) was used for all radiosynthetic runs in the present study.

[^11^C]Acetyl chloride ([^11^C]AcCl) was produced according to our previous procedure (Arai et al. 2009). Briefly, no-carrier-added [^11^C]CO_2_ was produced by the bombardment of dry N_2_ gas (1.5 MPa; Nippon Sanso, Japan) containing 0.01% O_2_ (Nippon Sanso) with a beam (15 mA) of 18 MeV protons. During the production of [^11^C]CO_2_, a solution of CH_3_MgBr (1.0 mol/L in THF, 500 μL) was loaded on the surface of a polyethylene loop, which was flushed with N_2_ to remove excess CH_3_MgBr solution.

After irradiation, [^11^C]CO_2_ was carried from the target with a N_2_ stream and trapped in a stainless-steel coil cooled to between − 170 °C and − 165 °C. The coil was warmed to 50 °C with hot air, and the concentrated [^11^C]CO_2_ was transferred to a cooled loop coated with the CH_3_MgBr solution until the level of radioactivity trapped in the loop reached a plateau. The radioactive mixture was transferred to the reaction vessel by passing oxalyl chloride (COCl)_2_/THF (10/400, 400 μL) through the loop, and heated at 50 °C for 2 min to produce [^11^C]AcCl.

The [^11^C]AcCl in the reaction vessel was distilled and trapped in a solution of **14** (1.0 mg), Et_3_N (5 μL), and DMAP (1 mg) in dry THF (300 μL). The reaction mixture was heated at 80 °C for 5 min. After the reaction, the reaction mixture was transferred to the injector for semipreparative HPLC. The preparative HPLC conditions were as follows: Triart C_18_ column (5 μm, 10 mm i.d. × 250 mm length, YMC), CH_3_CN/H_2_O/trifluoroacetic acid (TFA) (40/60/0.1%) as a mobile phase, a flow rate of 5.0 mL/min, and UV detection at 254 nm. The retention time (*t*_R_) of [^11^C]PK68 was approximately 7.5 min. The HPLC fraction of [^11^C]PK68 was collected in a flask to which polysorbate 80 (75 μL) in ethanol (0.3 mL) and ascorbic acid (25 mg/0.1 mL by injection) had been added before radiosynthesis. The solution was subsequently evaporated to dryness, and the residue was dissolved in physiological saline (5 mL). The resulting solution was passed through a Millex-GV filter (Millipore) to obtain [^11^C]PK68 as an injectable solution.

Radiochemical purity, identification, and molar activity were measured by analytical HPLC [mobile phase: CH_3_CN/H_2_O/TFA = 45/55/0.1%; flow rate: 1.0 mL/min; UV absorbance: 254 nm] using a Triart C_18_ column (4.6 mm i.d. × 250 mm length, YMC). The *t*_R_ was 5.0 min for [^11^C]PK68. The identification of [^11^C]PK68 was confirmed by co-injection with the corresponding unlabeled PK68. The mass (μmol) of [^11^C]PK68 with a known radioactivity (GBq) was determined by analytical HPLC comparison of the UV absorbance at 254 nm of [^11^C]PK68 with that of known concentrations of unlabeled PK68.

### Lipophilicity of [^11^C]PK68

The logD value was measured by mixing [^11^C]PK68 with n-octanol (3.0 g) and phosphate-buffered saline (PBS; 3.0 g, 0.1 M, pH 7.4) in a test tube, which was vortexed for 3 min at room temperature, then centrifuged at 3500 *g* for 5 min. An aliquot of 0.65 mL PBS and 0.65 mL n-octanol was removed and weighed, and its radioactivity was determined using an autogamma counter (2480 Wizard^2^, Perkin-Elmer, Waltham, MA, USA). Each sample from the remaining organic layer was removed and repartitioned until a consistent logD value was obtained. The logD value was calculated by comparing the ratio of counts per minute (cpm)/g of n-octanol to that of PBS, and is expressed as logD = log[cpm/g (n-octanol)/cpm/g (PBS)]. All measurements were performed in triplicate.

### Animal experiments

#### Animals

Male ddY mice were purchased from Japan SLC (Shizuoka, Japan), kept in a temperature-controlled environment under a 12 h light–dark cycle, and fed a standard diet (MB-1; Funabashi Farm, Chiba, Japan). The animal experiments were conducted according to the recommendations made by the Committee for the Care and Use of Laboratory Animals at the National Institutes for Quantum Science and Technology, and were approved by the Committee of National Institutes for Quantum Science and Technology (Approval Number: 16-1006).

#### Small-animal PET imaging

Each mouse was anesthetized using 1.5% (v/v) isoflurane, and an intravenous catheter was inserted into its tail vein. The mice (10–14 weeks old, 48.1 ± 7.4 g) were subsequently maintained under anesthesia and secured in a custom-designed chamber placed in the center of a small-animal PET scanner (Inveon, Siemens Healthineers, Erlangen, Germany). After adjusting the target position for scanning, dynamic emission scans (in three-dimensional list mode) were performed for 60 min (1 min × 4 frames, 2 min × 8 frames, and 5 min × 8 frames). [^11^C]PK68 (11–16 MBq) was injected via a tail vein catheter. The body temperature of each mouse was maintained at 37 °C using a heated (40 °C) water circulation system (T/Pump TP401, Gaymar Industries, NY, USA) during the PET scan. For the blocking study, the mice were pretreated with unlabeled PK68 (1 mg/kg) just before the injection of [^11^C]PK68. The obtained dynamic PET images (0.6 mm slice thickness) were reconstructed by filtered-back projection using a Hanning filter, with a Nyquist cutoff of 0.5 cycles per pixel. The time–activity curves (TACs) of [^11^C]PK68 were acquired using PMOD software (version 3.4, PMOD Technology, Zurich, Switzerland) from the volumes of interest, which were manually mapped onto the heart, lung, liver, kidney, small-intestine (part of duodenum), muscle, and brain. The radioactivity was decay-corrected to the injection time and is expressed as a standardized uptake value (SUV), normalized for injected radioactivity and body weight. The SUV was calculated according to the following formula: SUV = (radioactivity per milliliter tissue/injected radioactivity) × body weight (g). We also calculated the area under the curve (AUC) using TACs from 0 to 60 min.

### Metabolite analysis

Following intravenous injection of [^11^C]PK68 (37 MBq, 0.1 mL), the mice (8–9 weeks, 40.1 ± 5.3 g) were killed by cervical dislocation at 5, 15, 30, and 60 min. Blood and liver samples were obtained immediately. Each blood sample was centrifuged in a heparinized tube at 15,000 *g* for 2 min at 4 °C to separate the plasma. The supernatant was collected in a test tube containing CH_3_CN, and the resulting mixture was vortexed for 15 s, then centrifuged at 15,000 *g* for 2 min for deproteinization. The resulting supernatant was collected. Each liver sample was homogenized using a Silent Crusher S homogenizer (Rose scientific, Edmonton, Canada) in ice-cooled saline. The resulting homogenate was mixed with an equivalent amount of CH_3_CN, and centrifuged at 15,000 *g* for 2 min for deproteinization. An aliquot of the supernatant (0.1–1.0 mL) obtained from the plasma or liver homogenate was injected into the HPLC system with a radioactivity detector, and analyzed using a Capcell Pak C_18_ column (4.6 mm i.d. × 250 mm, Shiseido) with a mobile phase [CH_3_CN/H_2_O/TFA, (45/55/0.1, v/v/v)] at a flow rate of 1.0 mL/min. The percentage ratio of [^11^C]PK68 (*t*_R_ = 5.0 min) to total radioactivity (corrected for decay) on the HPLC chromatogram was calculated as % = (peak area for [^11^C]PK68/total peak area) × 100.

### Ex vivo biodistribution study

Each mouse (8 weeks old, 37.9 ± 1.0 g) was injected with a bolus of [^11^C]PK68 (2.7 MBq, 0.1 mL) via its tail vein. Three mice were sacrificed by cervical dislocation at each experimental time-point (1, 5, 15, 30, and 60 min) after the injection. The blood (heart contents), heart wall, lung, liver, pancreas, spleen, kidneys, adrenals, stomach (including contents), small intestine (including contents), large intestines (including contents), testes, muscles, and whole brain were removed quickly and weighed. The radioactivity in these tissues was measured with an autogamma scintillation counter (2480 Wizard^2^) and expressed as %ID/g. All radioactivity measurements were corrected for decay.

### Statistical analysis

Data are expressed as the mean ± standard deviation. Comparisons were made using two-way analysis of variance (ANOVA) with the Bonferroni post hoc test. The analysis was performed using GraphPad Prism 5 software (GraphPad Software, CA, USA). Differences between groups were considered significant when *P* <0.05.

## Results

### Chemistry

**Scheme 1.**
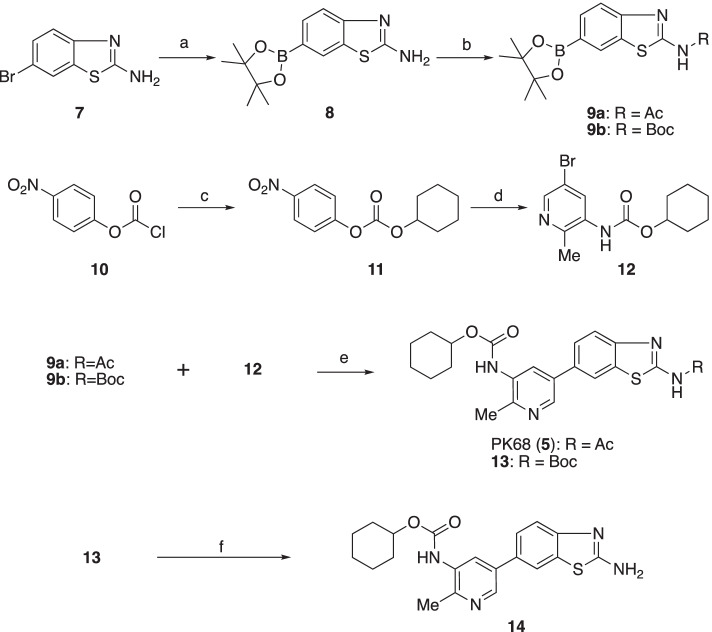
Chemical synthesis. Reagents and Conditions: **a** bis(pinacolato)diboron, Pd(dppf)_2_^⋅^CH_2_Cl_2_, K_2_CO_3_, 1,4-dioxane, reflux, 10 h; **b** Acetylchloride, Et_3_N, DMAP, CH_2_Cl_2_, room temperature, overnight, for **9a**; Boc_2_O, Et_3_N, DMAP, CH_2_Cl_2_, room temperature, overnight, for **9b**; **c** cyclohexanol, DMAP, THF, 0 °C, 3 h; **d** KN(TMS)_2_, toluene, 2-methyl-3-amino-5-bromopyridine in THF, 0 °C, 0.5 h; **e** Pd(Ph_3_P)_4_, K_2_CO_3_, 1,4-dioxane + H_2_O, reflux, 12 h; **f** 2 mol/L HCl, CH_3_OH, 60 °C, overnight

The standard sample PK68 and its ^11^C-labeling precursor **14** were obtained following the synthetic routes shown in Scheme 1. The procedures were performed according to the method described in the literature (Zhang et al. 2018b) with some modifications. The reaction of **8** with the corresponding acylating agents gave the intermediate amides **9a** and **9b** in 75% and 80% yields, respectively. Another carbamate intermediate **12** was obtained through the amidation of bicarbonate **11** with 2-methyl-3-amino-5-bromopyridine in 76% yield. PK68 and a *tert*-butoxycarbonyl (BOC) group-protecting intermediate **13** were obtained via a palladium-catalyzed cross-coupling reaction between **9a** or **9b** and **12** in 69% and 43% yields, respectively. The treatment of 2 mol/L HCl converted **13** to the ^11^C-labeling precursor **14** in 55% yield.

### Radiochemistry

Starting from 18–33 GBq of [^11^C]CO_2_, we prepared 1200–1790 MBq of [^11^C]PK68 with a radiochemical yield of 9.1 ± 5.9% (n = 10) (decay-corrected to the end of irradiation). The radiochemical purity of the [^11^C]PK68 solution was determined to be greater than 99%, with molar activity of 37–99 GBq/µmol. The radiochemical purity of [^11^C]PK68 remained greater than 95% after standing for 90 min at room temperature, indicating that this product was radiochemically stable for the period required for at least one PET scan. The lipophilicity (logD) of [^11^C]PK68 was 4.0, which was slightly over the range of moderate lipophilicity (2–3.5) for a PET tracer (Pike 2009).

### Small-animal PET imaging using mice

**Fig. 3:**
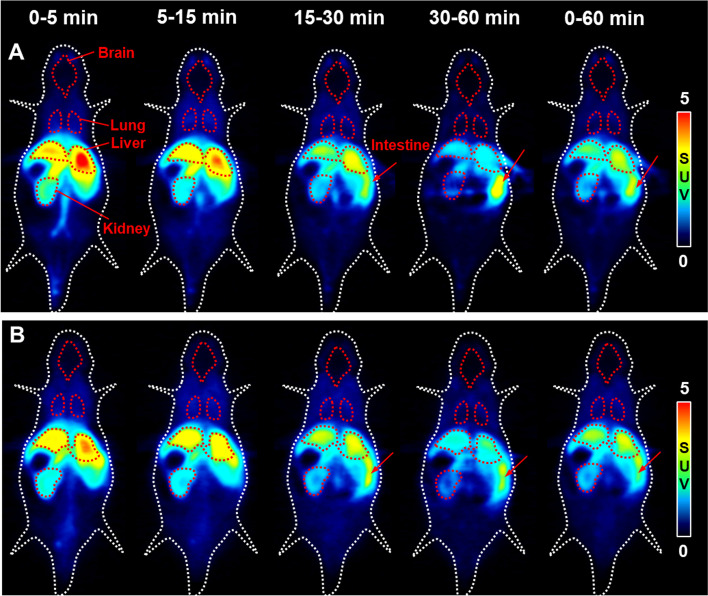
Representative dynamic (0–5, 5–15, 15–30, and 30–60 min) and summed (0–60 min) PET images of [^11^C]PK68 in mice treated without (baseline, n = 2, **A**) or with unlabeled PK68 (1 mg/kg, i.v., n = 2, **B**). Radioactivity in PET images expressed as standardized uptake values (SUVs)

To evaluate the in vivo kinetic and specific uptake of [^11^C]PK68, dynamic PET scans were conducted using mice treated with or without unlabeled PK68 at a concentration of 1 mg/kg. Figure 3 shows representative dynamic (0–5, 5–15, 15–30, and 30–60 min) and summed (0–60 min) PET images of the baseline (A) and blocking (B) mice (n = 2 in each group). In baseline mouse (A), the highest uptake of radioactivity in the initial phase (0–5 min) was observed in the liver, followed by the kidneys. These uptakes of radioactivity declined with time, whereas radioactivity in the intestine increased after the early phase (<15 min). The uptake of radioactivity by the other organs was low. Notably, the uptake of radioactivity by the brain was extremely low (<0.3 SUV). Following co-administration with unlabeled PK68, the radioactivity signals in the liver and kidney decreased slightly in the early phase (~ 15 min), which might imply specific binding to RIPK1 (Fig. 3b). On the other hand, accumulation of radioactivity in the intestine, a RIPK1-rich organ, was also decreased at late phase (>15 min) by the blocking. However, this phenomenon might be caused by competitive inhibition of metabolism rather than specific binding.

**Fig. 4:**
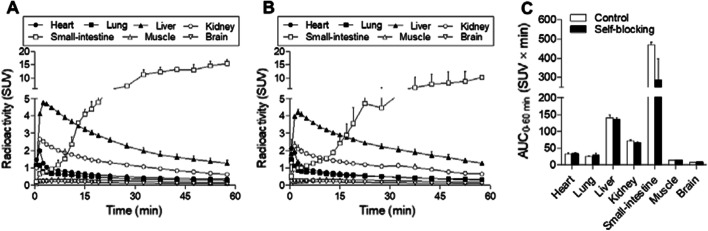
Time–activity curves (n = 2, in each group) of [^11^C]PK68 in the heart (buried circles), lung (buried squares), liver (buried triangles), kidney (open circles), muscle (open squares), and brain (open triangles) of mice treated without (**A**) or with unlabeled PK68 (1 mg/kg, i.v., **B**) and areas under the curves (**c**) in these tissues. Radioactivity is expressed as standardized uptake values (SUVs)

Figure 4 shows TACs of [^11^C]PK68 in the baseline (A) and blocking (B) studies and AUCs of radioactivity in the regions of interest (C). The radioactivity values in the liver and kidney peaked at 2.5 and 1.5 min after injection, and were 4.8 SUV and 2.7 SUV, respectively. By the blocking using unlabeled PK68, these radioactivity values declined to 4.2 SUV for the liver and 2.3 SUV for the kidneys. Although treatment with unlabeled PK68 reduced the maximum SUV values in these tissues by roughly 15%, there were no significant differences in the AUC values in these tissues between the groups because of small sample size (n = 2). Despite this fact, because the percentage of coefficient of variance was under 10% in almost tissues except for the small-intestine, the reproducibility of TACs in those tissues was very high.

### Metabolite analysis

**Fig. 5:**
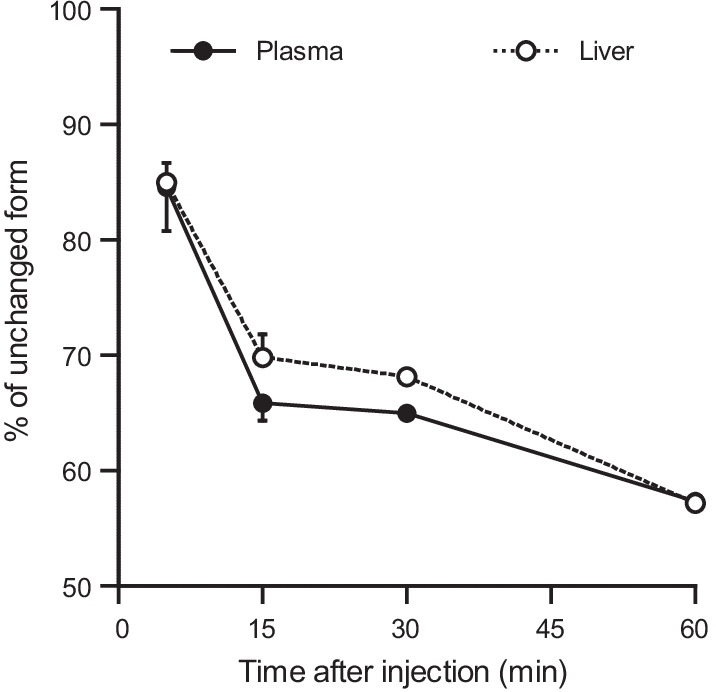
Unchanged form of [^11^C]PK68 in the plasma and liver of a mouse at 5, 15, 30, and 60 min after injection (n = 2)

Figure 5 shows the time-course of percentages of unchanged [^11^C]PK68 in the plasma and liver (n = 2 at each time-point). The fraction corresponding to [^11^C]PK68 (*t*_R_ = 5.0 min) in the plasma gradually decreased to 85% at 5 min, 66% at 15 min, 65% at 30 min, and 57% at 60 min after injection. A polar radiolabeled metabolite (*t*_R_ = 1.5 min) was observed in the plasma samples analyzed by HPLC (data not shown). Similarly, the levels of the unchanged form of [^11^C]PK68 in the liver were 85% at 5 min, 67% at 15 min, 68% at 30 min, and 57% at 60 min.

### Biodistribution of [^11^C]PK68 in the mice

To evaluate the distribution of [^11^C]PK68 in peripheral tissues, we performed ex vivo biodistribution study using mice. The radioactive concentrations (%ID/g tissue) of [^11^C]PK68 at various time-points (e.g., 1, 5, 15, 30, and 60 min) in each mouse tissue are shown in Table 1. At 1 min after injection of the radiotracer, there was high uptake (>5%ID/g) by the heart, lungs, liver, kidneys, and adrenals, which, with the exception of the liver, demonstrated the washout times from those organs. In the liver, the radioactivity decreased rapidly following an intense increment at 5 min after injection. However, the radioactivity in the small intestine increased with time, and reached a maximum level (>25%ID/g) 60 min after injection.

**Table 1:** Biodistribution of [^11^C]PK68 in mice (n = 3, mean ± SD)

Tissue	Time after the injection (min)				
	1	5	15	30	60
Blood	2.4 ± 0.3	1.3 ± 0.2	1.1 ± 0.1	0.8 ± 0.1	0.5 ± 0.1
Heart	7.6 ± 0.3	2.8 ± 0.3	2.1 ± 0.3	1.3 ± 0.1	0.7 ± 0.1
Lung	5.2 ± 0.2	2.6 ± 0.2	2.1 ± 0.3	1.5 ± 0.2	0.8 ± 0.1
Liver	8.0 ± 0.8	17.2 ± 0.7	15.6 ± 0.8	10.7 ± 0.3	6.0 ± 0.2
Pancreas	3.4 ± 0.2	2.7 ± 0.3	2.1 ± 0.3	1.4 ± 0.1	0.8 ± 0.1
Spleen	1.5 ± 0.2	1.6 ± 0.1	1.4 ± 0.2	0.9 ± 0.0	0.5 ± 0.1
Kidney	10.3 ± 0.5	9.9 ± 0.4	7.7 ± 1.0	6.2 ± 0.6	3.2 ± 0.1
Adrenals	6.2 ± 2.7	4.0 ± 0.5	3.3 ± 0.5	2.1 ± 0.8	1.2 ± 0.1
Stomach	1.2 ± 0.2	2.0 ± 0.1	4.0 ± 1.2	4.7 ± 1.0	7.2 ± 2.4
Small intestine	1.8 ± 0.1	3.3 ± 0.1	9.8 ± 3.1	16.7 ± 1.4	27.7 ± 3.0
Large intestine	0.7 ± 0.1	0.9 ± 0.1	1.4 ± 0.3	1.7 ± 0.4	2.6 ± 0.6
Testis	0.3 ± 0.1	0.3 ± 0.0	0.3 ± 0.0	0.3 ± 0.0	0.2 ± 0.0
Muscle	1.9 ± 0.3	1.1 ± 0.1	0.8 ± 0.1	0.6 ± 0.1	0.3 ± 0.0
Brain	0.6 ± 0.0	0.4 ± 0.0	0.3 ± 0.0	0.2 ± 0.0	0.1 ± 0.0

Radioactivity is expressed as %ID/g

## Discussion

RIPK1 is one of regulators involved in cellular apoptosis and necroptosis. Therefore, RIPK1, like COX-1/2 and TSPO, is considered as a target enzyme for treatment against cancers and other inflammation diseases in multiple tissues. PET imaging enables noninvasive evaluations of drug dynamics and estimations of drug occupancy for target molecule in vivo. Most recently, RIPK1 imaging in the brain has been conducted using [^18^F]CNY-07 (Fig. 2), an initial PET tracer for RIPK1 (Lan et al. 2021). The radioactive uptake of [^18^F]CNY-07 was very high (3%ID/cc) in the whole brains of healthy mice, and remained at high levels during the PET scan. However, the radioactive uptake of [^18^F]CNY-07 in the brain almost reflected nonspecific or off-target binding, but not related to RIPK1 binding, because of small decrease of brain uptake by the blocking. Therefore, to the best of our knowledge, until now there has been no useful PET tracer for imaging RIPK1.

In the present study, we selected PK68 (**5**) as the candidate PET imaging agent for RIPK1. PK68 has recently been identified as a potent inhibitor of RIPK1. It has high affinity for RIPK1 and exerts a strong inhibitory effect on it in human colon cancer (HT-29) and mouse fibroblast-like (L929) cells (Hou et al. 2019). Furthermore, melanoma (B16F10)-bearing mice treated with PK68 exhibit a significant reduction in the number of tumor metastases that is the same as in B16F10-bearing mice treated with Necrostatin-1 (**1**).

**Scheme 2.**
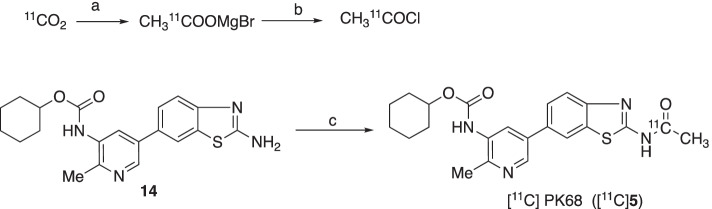
Radiosynthesis. Reagents and conditions: **a** 1 mol/L CH_3_MgBr in THF; **b** (COCl)_2_, 2,6-di-*tert*-butylpyridine in DMF; **c** [^11^C]acetyl chloride ([^11^C]AcCl), Et_3_N, DMAP, THF, 80 °C, 5 min

We initially decided to label PK68, which has an unsymmetrical urea moiety, using [^11^C]phosgene (Ogawa et al. 2010). However, we failed to obtain [^11^C]PK68 and found that symmetrical cyclohexyl [^11^C]bicarbonate was a major product. We then labeled PK68 using [^11^C]AcCl, and successfully obtained [^11^C]PK68 with enough radioactivity (>1200 MBq and >3% of radiochemical yield) for an animal evaluation study (Scheme 2). The [^11^C]PK68 in the saline-formulated solution maintained 95% radiochemical purity after 90 min at room temperature, indicating it would be radiochemically stable for the duration of one PET scan.

In PET imaging using mice, the brain uptake of [^11^C]PK68 was unfortunately inferior to that of [^18^F]CNY-07, and the maximum uptake in the brain was under 0.25 SUV (<1%ID/cc) (Figs. 3a, 4a). It has been suggested that, to some extent, the permeability of the brain with regard to [^11^C]PK68 may be restricted by ATP-binding cassette transporters at the brain-blood barrier. Or, [^11^C]PK68 might readily bind to plasma protein owing to its high lipophilicity, impeding entry into the brain.

RIPK1 is constitutively expressed both in the brain and in many peripheral organs (Zhang et al. 2010). Herein, we evaluated the dynamics of [^11^C]PK68 in the peripheral organs in vivo. In the PET imaging and biodistribution studies on healthy mice, [^11^C]PK68 rapidly accumulated in the liver and kidneys, and subsequently its radioactive metabolites were distributed in the small intestine (Fig. 3a and Table 1). Such dynamics indicate typical hepatobiliary excretion and the intestinal reuptake pathway, which dominates the whole-body distribution of radioactivity and rapid washout from the body after the injection. In this manner, we successfully demonstrated the biodistribution of PK68 in mice by PET imaging and dissection. This will improve our understanding of the kinetics of PK68 derivatives for the further development of PET tracer candidates for imaging RIPK1. To confirm the specific binding of [^11^C]PK68 for RIPK1 in the peripheral organs, we also performed a blocking study by pretreatment with unlabeled PK68 (1 mg/kg). As shown in the PET images, radioactive uptake in the liver was reduced by the blocking study in the initial phase (0–5 min) after the injection (Fig. 3a). Although the differences between the control and blocking subjects with regard to the AUC values of the liver were insignificant (Fig. 4c), a slight reduction in radioactive uptake by the liver owing to the blocking study might have corresponded with the specific binding of [^11^C]PK68 with RIPK1. The metabolite analysis revealed that the level of the unchanged form of [^11^C]PK68 was slightly higher in the liver than in the plasma until 30 min after the injection (Fig. 5). These results imply that radioactive uptake by the liver may involve the specific binding of [^11^C]PK68 with RIPK1. It was known that expression of RIPK1 in the liver was upregulated in inflammation and cancer (Kondylis and Pasparakis 2019). Since inflammation reaction daily occurs in the liver with small level even under the healthy condition, RIPK1 in liver of healthy mouse would exist with nonnegligible level.

Interestingly, the metabolite rate of [^11^C]PK68 in the plasma were relatively slow, and the level of the unchanged form of [^11^C]PK68 remained >50% 60 min after the injection. [^11^C]PK68 labeled with the [^11^C]acetyl group would be more stable in vivo than some useful PET tracers labeled with the conventional [^11^C]methyl group, such as [^11^C]DAA1106 (Zhang et al. 2003), [^11^C]AC-5216 (Zhang et al. 2007), [^11^C]MeNER (Schou et al. 2009), [^11^C]DAC (Yanamoto et al. 2009), and [^11^C]ITDM (Bertoglio et al. 2020). This is one of favorable profiles as a PET tracer for imaging target molecules. Together, [^11^C]PK68 may enable the visualization of upregulated RIPK1 in the peripheral organs under pathological conditions, although the current work using healthy animals did not reveal significant specific binding to RIPK1. Moreover, PET with [^11^C]PK68 would give useful information about design of chemical structure for the further development of PET tracer candidates.

## Conclusions

In the present study, we successfully synthesized [^11^C]PK68 as a potential PET tracer for RIPK1. However, PET imaging revealed no obvious specific binding of [^11^C]PK68 for RIPK1 in healthy mice. Although [^11^C]PK68 exhibited poor permeability into the brain, it may enable the visualization of RIPK1 if an inflammation model of peripheral organs is used. In future experiments, we will investigate the specific binding of [^11^C]PK68 with RIPK1 in PET studies using pathological animal models, such as liver inflammation, peripheral ischemia, and tumor-bearing models. We will also progress optimization of the chemical structures of promising candidates for imaging RIPK1.

## Data Availability

Data can be obtained upon request.

## Abbreviations

[^11^C]AcCl: [^11^C]acetyl chloride
AUC: Area under the curve
ESI–MS: Electrospray ionization–mass spectrometry
PK68: Cyclohexyl (5-(2-acetamidobenzo[*d*]thiazol-6-yl)-2-methylpyridin-3-yl) carbamate
mpt: Melting point
HRMS: High-resolution fast atom bombardment mass spectra
%ID/g: Percentage of injection dose per gram tissue
PET: Positron emission tomography
RIPK1: Receptor-interacting protein 1 kinase
*t*_R_: Retention time
SUV: Standardized uptake value
TMS: Tetramethylsilane
TAC: Time-activity curve
TFA: Trifluoroacetic acid
